# Draft Genome Sequence of *Ensifer adhaerens* M78, a Mineral-Weathering Bacterium Isolated from Soil

**DOI:** 10.1128/genomeA.00969-16

**Published:** 2016-09-08

**Authors:** Yuanli Wang, Wei Chen, Linyan He, Qi Wang, Xia-Fang Sheng

**Affiliations:** Key Laboratory of Agricultural and Environmental Microbiology, Ministry of Agriculture, College of Life Sciences, Nanjing Agricultural University, Nanjing, People’s Republic of China

## Abstract

*Ensifer adhaerens* M78, a bacterium isolated from soil, can weather potash feldspar and release Fe, Si, and Al from rock under nutrient-poor conditions. Here, we report the draft genome sequence of strain M78, which may facilitate a better understanding of the molecular mechanism involved in mineral weathering by the bacterium.

## GENOME ANNOUNCEMENT

The weathering of rocks is an important environmental process, and many studies have shown that bacteria play an important role in it (1, 2). *Ensifer adhaerens* strain M78, a mineral-weathering bacterium, was isolated from soil in Sichuan, China. A mineral-weathering experiment showed that strain M78 was able to release significantly more Fe, Si, and Al from potash feldspar by producing organic acids and exopolysaccharides. Hence, the genome of this organism was sequenced to obtain better insights into the molecular mechanism of mineral weathering by this strain.

The genome of strain M78 was extracted and purified following methods reported previously (3). The purified genome was then sequenced using Illumina MiSeq sequencing technology by the Shanghai BIOZERON Biotechnology Co., Ltd. (Shanghai, China). The adapter, poly-N, error, and low-quality reads and small fragments (length <50 bp) were removed from the raw reads, generating 5,449,814 high-quality paired-end reads and resulting in a 195-fold depth of coverage. High-quality reads were then assembled using SOAPdenovo version 2.04 (http://soap.genomics.org.cn). Segmental gaps were filled and bases were corrected by GapCloser version 1.12 software. Genome annotation was carried out in the NCBI Prokaryotic Genome Annotation Pipeline (4). Functional classification was performed by aligning predicted proteins to the Clusters of Orthologous Groups database (5).

The draft genome sequence of *Ensifer adhaerens* M78 was 6,937,494 bp in length with an average G+C content of 62.2%. All generated reads were assembled into 65 scaffolds with an *N*_50_ length of 239,007 bp. Gene prediction for strain M78 was carried out using Glimmer version 3.02 (http://ccb.jhu.edu/software/glimmer/index.shtml), which predicted the presence of 6,642 candidate protein-coding genes, giving a coding intensity of 85.1%; the average length of each gene is 888 bp. The genome was shown to encode nine rRNAs and 62 tRNAs, along with 208 predicted metabolic pathways.

According to the annotation results, many genes and metabolic pathways (signal transduction mechanisms, cell motility, and carbohydrate transport metabolism) may be involved in mineral weathering. Moreover, some genes encoding organic acids and exopolysaccharide biosynthesis were found in our genome sequence; these organic acids and exopolysaccharides play important roles in the mineral weathering process (6, 7). Furthermore, there are 424 genes with unknown functions, which may be correlated with mineral weathering.

### Accession number(s).

This whole-genome shotgun project has been deposited at DDBJ/ENA/GenBank under the accession number MAUG00000000. The version described in this paper is the first version, MAUG01000000.
